# The impact of adverse childhood experiences and posttraumatic stress symptoms on chronic pain

**DOI:** 10.3389/fpsyg.2023.1243570

**Published:** 2023-11-17

**Authors:** Peta Stapleton, Yage Kang, Robert Schwarz, John Freedom

**Affiliations:** ^1^School of Psychology, Bond University, Gold Coast, QLD, Australia; ^2^The Association for Comprehensive Energy Psychology, Bryn Mawr, PA, United States

**Keywords:** chronic pain, pain interference, pain intensity, adverse childhood experiences, post traumatic stress symptoms (PTSS)

## Abstract

**Introduction:**

Chronic pain is a prevalent worldwide health condition. The current study aimed to extend previous research that investigated the dynamics between chronic pain, adverse childhood experiences (ACEs), and post-traumatic stress symptoms (PTSS).

**Method:**

Adult participants worldwide with chronic pain were recruited for this study (*N* = 199; 89% females). Three hypotheses were proposed: (a) a high ACEs score would result in more severe pain intensity and interference compared to no ACEs; (b) a low ACEs score would result in more severe pain intensity and interference compared to no ACEs; and (c) PTSS would fully mediate the ACEs-pain relationship.

**Results:**

Initially results indicated individuals with high ACEs reported more pain interference than those with no ACEs, although pain intensity did not differ between high and no ACEs. However, after controlling for age, socioeconomic status (SES), and pain duration, low and high ACEs were not significantly associated with pain intensity or interference compared to no ACEs. However, SES status was associated with pain intensity and interference, although not with pain interference after adding low and high ACEs to the model. Because of this the mediation exploration of PTSS was not viable.

**Discussion:**

Implications for practice, limitations and future research outcomes are outlined.

## The impact of adverse childhood experiences and posttraumatic stress disorder on chronic pain

Chronic pain is a pervasive health condition that refers to ongoing and persistent pain experienced over three months without definitive medical explanations (Treede et al., 2015). The effective treatment of chronic pain is essential, as it can significantly interfere with individuals’ lives, such as work capacity, emotional well-being, and overall quality of life, and result in productivity losses for society (Tidmarsh et al., 2022). The global prevalence of chronic pain is estimated to affect between a third to 50% of the population (Johannes et al., 2010; Katz et al., 2015; Treede et al., 2015; Dahlhamer et al., 2018). Pharmacological treatments, including analgesics, are commonly prescribed as first-line treatments for chronic pain to alleviate symptom severity and improve functioning (Kuijpers et al., 2011). However, long-term use of these medications can result in adverse effects such as side effects, addiction and tolerance. The efficacy of medication alone may also be limited due to the contribution of psychological factors, to the development and maintenance of pain. One such risk factor includes negative early life experiences or exposure to adverse childhood experiences (ACEs; Tidmarsh et al., 2022). ACEs are defined as potentially traumatic encounters before 18 years of age (Tidmarsh et al., 2022). These include physical, psychological, emotional, and/or sexual abuse or neglect by a primary caregiver or parental psychopathology, witness to violence against a child’s mother, living with substance abuse, and early parent loss (Felitti et al., 1998). It is thought these early life experience(s) may influence chronic pain occurrence and presentation in adulthood.

Gatchel (2004), Gatchel et al. (2007), and Gatchel and Turk (2008) proposed a conceptual framework, the Biopsychosocial Model of Pain, that has been examined for the potential impact of ACEs on the development of chronic pain (Nelson et al., 2021). It is postulated that ACEs may alter the biological stress response system, resulting in *allostatic overload* (Christensen et al., 2021; Nelson et al., 2021). Allostatic overload refers to a cumulative physiological process in which repeated exposures to stressful events may lead to the excessive release of stress hormones over time, and maladaptive activation of the hypothalamic-pituitary-adrenal (HPA) axis, ultimately causing physical complications such as cardiovascular problems (e.g., heart rate and blood pressure), inflammation, and metabolic processes (e.g., cholesterol and insulin; Su et al., 2015). A recent systematic review of biomarker outcomes supported the relationship between ACEs and allostatic overload, demonstrating that individuals with higher ACEs exhibited more significant physiological dysregulation (Finlay et al., 2022).

Previous research on the relationship between ACEs and pain has suggested that childhood trauma may increase inflammation and sensitize pain pathways, thereby contributing to the onset of chronic pain and perpetuating the pain through a cycle of pain and stress (Christensen et al., 2021). However, the literature on ACEs and chronic pain has yielded inconsistent and conflicting findings. For instance, Groenewald et al. (2020) reported that among 48,567 children aged six to 17, who experienced one ACEs had a 60% increased risk of developing chronic pain compared to children who experienced no ACEs, while those with four or more ACEs faced a 170% increased risk. ACEs have also been linked to various types of chronic pain in adulthood, such as arthritis (Von Korff et al., 2009), functional somatic syndrome (Davis et al., 2005), and chronic back and neck pain (Stickley et al., 2015). However, a meta-analysis conducted by Marin et al. (2021) reported weak to no association between ACEs and chronic pain, concluding that the ACEs-pain relationship has not been firmly established by research. It was also reported that ACEs did not predict pain outcomes, specifically interference or pain intensity (Häuser et al., 2019).

What is evident is that not all forms of ACEs seem to be associated with chronic pain in adulthood; rather ACES should be severe enough to trigger allostatic overload, which may then cause inflammations and sensitize the pain pathways (Beal et al., 2020; Kaleycheva et al., 2021). Consequently, some researchers argue that ACEs should be severe enough to cause posttraumatic stress disorder (PTSD) to impact pain pathways, since PTSS often result from overactivated and maladaptive biological stress responses (Beal et al., 2020; Nelson and Cunningham, 2020; Nelson et al., 2021). Studies have suggested PTSD and maltreatment ACEs together predicted increased chronic pain risk and more severe pain outcomes (Raphael and Widom, 2011), and PTSD as a diagnosis either fully or partially mediated the relationship between ACEs and pain outcomes (McKernan et al., 2019; Beal et al., 2020). However, it is unclear as to the level of ACEs exposure (e.g., dose) that triggers this allostatic overload, and the relationship to PTSS.

Therefore, the present study aimed to examine the level of ACE exposure, categorized as: no ACEs, low ACEs (one to three incidents), and high ACEs (four to 10 incidents). This classification was based on previous findings suggesting that one to three ACEs may result in significantly more chronic pain compared to no ACES (Groenewald et al., 2020; Alhowaymel et al., 2023), and four or more ACEs resulted in a significant increase in risk of chronic pain and PTSS compared to low or no ACEs (Nelson et al., 2021; Alhowaymel et al., 2023). The following hypotheses were proposed:

(1) It was hypothesized that high ACEs would lead to more severe pain intensity and interference (a chronic pain profile) compared to no ACEs.
(2) Low ACEs would lead to more severe pain interference and pain intensity compared to no ACEs.
(3) PTSS would fully mediate the relationship between ACEs and pain outcomes.

A qualitative investigation of themes in an open-ended question about the nature of participants’ chronic pain was also examined for a wider intervention trial offered (not reported here).

## Materials and methods

### Participants

There were 323 participants recruited via community advertising on social media sites, through general practitioners specializing in pain, and on online noticeboard, for a study investigating the efficacy of a psychological intervention for chronic pain (see Stapleton et al., 2022). Due to the nature of the intervention study being focused on medically unexplained chronic pain, the inclusion and exclusion criteria was strict (see below).

An *a priori* power analysis was conducted for a mediation analysis using G*Power 3.1.9.4 program (Faul et al., 2009). The power analysis revealed that a minimum sample of 138 participants was necessary for securing a power of 0.95 for a moderate effect size of 0.15, using an alpha of 0.05 (Cohen, 1988). A total of 124 participants were deleted due to measurement incompletion, leaving 199 participants in the data analysis for the current study which was appropriate for the intended analysis. The gender distribution in the sample (*N* = 199) included 177 females, 21 males, and one person identified as non-binary, making up 88.9%, 10.6%, and 0.5% of the sample, respectively. The age of the participants ranged from 18 to 80 (*M* = 53.94, *SD* = 12.57). The reported pain duration from the participants ranged from three months to 66 years (*M* = 12.12, *SD* = 11.78). Demographic information is presented in Table 1.

**TABLE 1:** The demographic characteristics of participants.

	*n*	%
Gender		
Female	177	88.9
Male	21	10.6
Other	4	0.5
Marital status		
Single	20	10.1
Married/partnered	40	20.1
Divorced/separated	138	64.3
Widowed	11	5.5
Household occupancy		
One	53	26.6
Two	83	41.7
Three	23	11.6
Four or more	40	20.1
Highest education level		
Highschool	24	12.1
Vocational/tech college	38	19.1
Bachelors	65	32.7
Masters	34	17.1
Doctoral	9	4.5
Others	29	14.6
Employment		
Unemployed	19	9.5
Student	12	6.0
Employed	133	66.9
Retired	35	17.6
Household income		
<10k	13	6.5
10–30k	34	17.1
30–50k	30	15.1
50–70k	29	14.6
70–90k	28	14.1
90–110k	22	11.1
>110k	43	21.6

N = 199. Participants were on average 53.94 years old (SD = 12.57). The pain duration was on average 12.12 years (SD = 11.78).

#### Inclusion and exclusion criteria

Participants needed to be at least the age of 18 to participate in the study. Participants needed to experience chronic pain for a minimum of three months in the past year, negatively impacting their overall functioning and quality of life. The types of chronic pain need to be determined as medically unexplained by health professionals (e.g., amyotrophic lateral sclerosis). The pain rating had to be at least four out of 10 on the Visual Analog Pain Index. Presence of psychiatric disorders (e.g., bipolar disorder) and co-morbid autoimmune diseases (e.g., rheumatoid arthritis) are exclusion criteria. The pain could not be malignant pain caused by cancer pain syndrome. Individuals were also excluded from the study if they were engaging with other psychological interventions for chronic pain, enrolling in high-intensity substance use treatment programs, or planning or receiving cancer treatment with radiation or chemotherapy at the time of the current research.

### Measures

The assessment package included an explanatory statement, a consent form, a demographic questionnaire, and three assessment measures. It took approximately 15 min to complete the package. The internal consistency reliability of each measurement was performed on our sample to ensure reliability.

#### Brief pain inventory

The BPI is a widely used self-report instrument to assess pain outcomes utilizing two subscales: Pain Severity and Pain Interference (Cleeland, 1991). The Pain Severity subscale consists of four items that measure the intensity of the pain (e.g., “Please rate your pain by selecting the number that best describes your pain on the average”). The Pain Interference subscale includes seven items that assess the extent to which pain interferes with various aspects of daily life (e.g., “Pain has interfered with your general activity”). Responses are given on an 11-point Likert scale, ranging from 0 (no pain/does not interfere) to 10 (pain as bad as you can imagine/completely interferes). The total scores can range from 0 to 40 for severity and 70 for interference, with higher scores indicating more severe intensity or interference. The BPI demonstrated high internal consistency in the current sample (Cronbach’s alpha = 0.92).

#### Adverse childhood experience questionnaire

The ACEsQ is a self-report questionnaire used widely to assess the impact of adverse childhood experiences on health and well-being in adulthood (Felitti et al., 1998). The ACES questionnaire comprises 10 items assessing exposure to different forms of abuse, neglect, and household dysfunction before age 18. Responses are given on a binary scale (yes or no) to each item (e.g., “Did a household member go to prison?”). The score range for the ACEs questionnaire is 0 to 10, with higher scores indicating greater exposure to adverse experiences. The ACEs demonstrated good internal consistency reliability on the current sample (Cronbach’s alpha = 0.80).

#### The two-item abbreviated post-traumatic stress disorder screener

The two-item PTSD screener developed by Lang and Stein (2005) is a brief self-report questionnaire used to screen for two core symptoms of PTSD: intrusion and hyperarousal. The first item asks participants to rate how often they had repeated and disturbing thoughts of a stressful experience. The second item assesses whether they would become upset when something reminded them of the stressful experience. The responses were given on a 5-point Likert scale, ranging from 0 (not at all) to 4 (extremely). The total score ranges from 0 to 8, with a score of three or higher suggests further investigation of probable PTSD. The scale showed high internal consistency reliability on the current sample (Cronbach’s alpha = 0.88). This measured was included to examine PTSS and not for diagnosis.

See Table 2 for the means and standard deviations for the above measures.

**TABLE 2:** Means and standard deviations for measures.

	*N*	Minimum	Maximum	Mean	Std. deviation
ACE total	199	0	10	7.04	2.57
Intensity total	199	0	39	20.96	6.29
Interference total	199	0	70	39.57	15.71
PTSD screener	197	2	8	4.92	2.17

### Procedure

The research first gained ethical approval from the Bond University Human Research Ethics Committee. The assessment package was then created on an external web-based survey tool, Psychdata.^1^ Participants were recruited using community advertisements that provided a study summary and a link to register if they were interested in participating. Once registered, participants were emailed a link to the Psychdata study, presenting the explanatory statement that provided further information about the study and were required to provide written consent. They then completed the assessment battery and proceeded to participate in the psychological intervention (see Stapleton et al., 2022). The data relating to the intervention trial was not analyzed in the current study.

The research used a correlational design to investigate the relationship between ACEs, PTSS, and pain outcomes. ACEs were split into three categories: no ACEs (score of 0 on ACEs Questionnaire), low ACEs (score ranging from one to three) and high ACEs (score ranging from four to 10). There were two outcome variables: pain intensity and pain interference measured by the BPI. The mediating variable was to be PTSS, measured by the two-item PTSD screener. Data was analyzed using the computer program Statistical Package for Social Science (SPSS Version 26.0) for dummy-coded linear regression and Jeffrey’s Amazing Statistics Program (JASP) for mediation. The qualitative data was analyzed with frequency analysis and thematic analysis with nVivo. Regression was employed for the quantitative analysis and the assumptions were conducted on all continuous variables. The assumption of homoscedasticity and normality of the residuals were met, as assessed by the visual inspection of the P-P plot.

## Results

A frequency analysis was conducted to examine the frequencies of endorsed ACEs items by the participants to determine which ACEs items are more commonly reported than others. The result was presented in Table 3.

**TABLE 3:** Results from the frequency analysis of the ACEs items.

	Frequency	Percentage	General descriptions
Item 1	88	44.2	Emotional abuse
Item 2	62	31.2	Physical abuse
Item 3	73	36.7	Sexual abuse
Item 4	93	46.7	Invalidation
Item 5	21	10.6	Neglect
Item 6	64	32.2	Parental separation
Item 7	24	12.1	Physical abuse
Item 8	65	32.7	Familial substance use
Item 9	90	45.2	Familial mental health
Item 10	11	5.5	Familial prison history

N = 199.

### Qualitative analysis

Qualitative data collected from the single open-ended question about the nature of chronic pain (*N* = 199) was transferred to NVivo for thematic analysis using a method of inductive reasoning (Schulz, 2012). Quantitative data was imported to SPSS and JASP for analysis.

#### Inductively developed thematic categories

**FIGURE 1:**
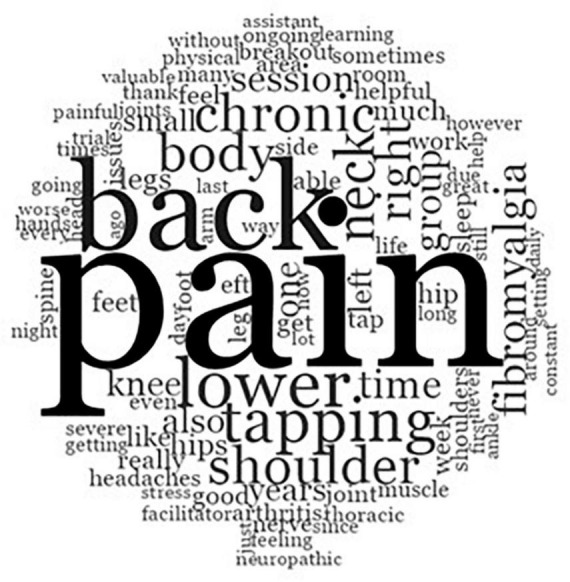
Sources of pain identified by participants.

Text search usage and word frequency data analyses determined emerging themes that were elicited by the qualitative question and are graphically displayed in a word cloud. As illustrated in Figure 1, larger texts indicate more frequently reported themes. Three central themes emerged: pain, back and lower. These themes were incorporated in the wider intervention trial.

### Quantitative analysis

A dummy-code regression was utilized to investigate the effect of different doses of ACEs on pain intensity and interference. The multivariate collinearity was checked using the Mahalnobis distance (21.91), which was smaller than the value of 22.46 (*df* = 6, *p* = 0.001). The assumption of the linearity was checked by the visual inspection of the P-P plot and the superimposed regression line was plotted. It is important to note that a slight floor effect was observed for the ACEs variable, specifically many participants scored 0. This was consistent with the research, which showed that approximately 50% of population may have no ACEs (Yau et al., 2022). ACEs was also split into three categories, which prevented them from the violation of linearity. Hence, the regression could continue.

The results revealed that there was no significant relationship between low ACEs and pain intensity β = 0.56, *SE* = 1.18, *t*(199) = 0.47, *p* = 0.641, or pain interference β = 3.06, *SE* = 2.93, *t*(199) = 1.05, *p* = 0.297. High ACEs were not significantly associated with pain intensity β = 1.66, *SE* = 1.18, *t*(199) = 1.40, *p* = 0.162. However, there was a significant relationship between high ACEs and interference β = 6.23, *SE* = 2.93, *t*(199) = 2.13, *p* = 0.035. The effect size was small to medium (Cohen’s d = 0.35). The means and standard deviation of the groups were reported in Table 4. The results indicated that compared to no ACEs, low and high ACEs were not significantly associated with pain intensity. Because of the wide range of ages (18 to 80 years), the vast majority of the sample being female, and divorcees, age, socioeconomic status, and pain duration were then controlled for in the regression.

**TABLE 4:** The mean and standard deviation of pain intensity and interference by ACEs groups.

Measure	No ACEs			Low ACEs			High ACEs		
	*n*	*M*	SD	*n*	*M*	SD	*n*	*M*	SD
Pain intensity	45	20.11	7.16	77	20.66	6.10	77	21.77	5.91
Pain interference	45	35.98	18.11	77	39.04	15.29	77	42.21	14.30
PTSS	45	4.18	2.01	77	4.59	2.01	77	5.68	2.20

N = 199.

These results then revealed that there was no significant relationship between low ACEs and pain intensity β = 0.37, SE = 1.18, *t*(199) = 0.31, *p* = 0.754, or pain interference β = 2.80, SE = 2.92, *t*(199) = 0.96, *p* = 0.338. High ACEs did not result in more pain intensity β = 1.26, SE = 1.19, *t*(199) = 1.07, *p* = 0.288, or pain interference β = 5.47, SE = 2.95, *t*(199) = 1.86, *p* = 0.065. The results indicated that after controlling for age, SES (income level), and pain duration, low and high ACEs were not significantly associated with pain intensity or interference compared to no ACEs (*p* = 0.065). However SES related to income (*M* = 4.32, SD- = 1.96) was associated with pain intensity in model 1 β = −0.55, SE = 0.23, *t*(199) = −2.44, *p* = 0.016 and model 2 β = −0.52, SE = 0.23, *t*(199) = −2.25, *p* = 0.025. SES was also associated with pain interference in model 1 β = −1.18, SE = 0.57, *t*(199) = −2.30, *p* = 0.040, but not in model 2 β = −1.03, SE = 0.57, *t*(199) = −1.80, *p* = 0.073. SES was not associated with pain interference after adding low and high ACEs to the model. See Tables 5, 6 for the associations.

**TABLE 5:** Regression of association between pain intensity by ACEs groups.

		Unstandardized coefficients		Standardized coefficients			95.0% Confidence interval for B	
Model		B	Std. error	Beta	t	Sig.	Lower bound	Upper bound
1	(Constant)	25.348	2.300		11.021	<0.001	20.812	29.883
	Total annual income	-0.554	0.228	-0.173	-2.436	0.016	-1.003	-0.105
	Age	-0.033	0.037	-0.066	-0.903	0.367	-0.105	0.039
	Length of concern	-0.017	0.039	-0.032	-0.442	0.659	-0.095	0.060
2	(Constant)	24.367	2.541		9.590	<0.001	19.356	29.378
	Total annual income	-0.519	0.230	-0.162	-2.254	0.025	-0.973	-0.065
	Age	-0.029	0.037	-0.058	-0.791	0.430	-0.102	0.043
	Length of concern	-0.019	0.039	-0.035	-0.477	0.634	-0.097	0.059
	Low ACE	0.369	1.176	0.029	0.313	0.754	-1.952	2.689
	High ACE	1.264	1.187	0.098	1.065	0.288	-1.077	3.604

a. dependent variable: intensity.

**TABLE 6:** Regression of association between pain interference by ACEs groups.

		Unstandardized coefficients		Standardized coefficients			95.0% Confidence interval for B	
Model		B	Std. error	Beta	t	Sig.	Lower bound	Upper bound
1	(Constant)	51.994	5.741		9.057	<0.001	40.672	63.316
	Total annual income	-1.177	0.568	-0.147	-2.072	0.040	-2.297	-0.057
	Age	-0.112	0.091	-0.090	-1.226	0.222	-0.292	0.068
	Length of concern	-0.110	0.098	-0.081	-1.116	0.266	-0.303	0.084
2	(Constant)	47.397	6.307		7.515	<0.001	34.957	59.837
	Total annual income	-1.028	0.571	-0.129	-1.800	0.073	-2.155	0.098
	Age	-0.096	0.091	-0.077	-1.053	0.294	-0.276	0.084
	Length of concern	-0.117	0.098	-0.087	-1.197	0.233	-0.310	0.076
	Low ACE	2.804	2.920	0.087	0.960	0.338	-2.955	8.564
	High ACE	5.468	2.946	0.170	1.856	0.065	-0.342	11.277

a. dependent variable: interference.

Because neither low ACEs or high ACEs were associated with pain interference or intensity, a meditation analysis of the impact of PTSS was not conducted.

## Discussion

The purpose of the current research was to build upon and extend previous findings by focusing on the post traumatic stress symptoms of intrusion and hyperarousal and their potential role in mediating the ACEs-pain relationship. However, because of the non-significant association of low or high ACEs on pain interference and intensity, a meditation was not conducted.

The findings of the current study did not support the first hypothesis as there was no association between high ACEs and pain intensity or interference. These results are inconsistent with prior research which has indicated that individuals with high ACEs experience greater pain-induced interference and functional disabilities in their daily lives (Beal et al., 2020; Nelson and Cunningham, 2020). In addition, other studies suggest that individuals with high ACEs also exhibit higher pain intensity (McKernan et al., 2019; Kaleycheva et al., 2021). The results provide evidence for the view that pain interference and intensity may have different mechanisms underlying their maintenance (Rogers and Farris, 2022). Since the current research utilized a sample of middle-aged and older adults (*M* = 53.94; *SD* = 12.57) with a long period of chronic pain (*M* = 12.12; *SD* = 11.78), the onset of injury leading to chronic pain might have occurred decades ago; pain intensity and interference may have been managed with other coping mechanisms (Sorensen et al., 2016).

However, SES level (specifically the $50 000 - $70 000 bracket) was associated with pain intensity and interference, although this became non-significant after adding low and high ACEs to the model. Previous research indicates a moderate increase in the risk of chronic pain for low and medium SES when compared with high SES (Prego-Domínguez et al., 2021), likely due to the increased chronic stress and/or PTSS and ACEs being associated with lower SES and/or the increased chance of work-related injury in low SES jobs (Mechlin, 2012; Kraft and Kraft, 2021). This sample could be considered moderate income, and was mostly educated (54% having a Bachelor’s degree or higher) and reported lengthy pain (12 + years). It appears there was still self-perceived financial hardship which may have been impacting daily pain intensity and/or interference. Intervention programs may need to take into account the impact of chronic pain for moderate income patients as they too may be associated with poorer health outcomes (Atkins and Mukhida, 2022).

The results did not support the second hypothesis; specifically, there was no significant difference in pain intensity and interference between the low and no ACEs groups. Despite the lack of statistical significance, these findings were consistent with previous research reporting that ACEs was not associated with pain outcomes (Häuser et al., 2019; Marin et al., 2021). One possible explanation for the result may be that the ACEs questionnaire did not distinguish maltreatment ACEs from general childhood adversity. For example, the frequency analysis revealed the most rated item in the ACEs questionnaire was “Your family did not look out for each other, feel close to each other, or support each other”. This item captured emotional neglect as an ACEs; however, it may not be severe enough to result in allostatic overload and affect pain outcomes compared to items directly related to physical or sexual abuse (Beal et al., 2020). The individuals in the low ACEs group may have endorsed similar low-severity items in the ACEs questionnaire, which did not elicit an effect on pain outcomes.

The current sample was comprised of middle to older adults (mean age 53 years), and although ACEs have been linked to various types of chronic pain in adulthood, such as arthritis (Von Korff et al., 2009), functional somatic syndrome (Davis et al., 2005), and chronic back and neck pain (Stickley et al., 2015), others have found ACEs may not predict pain outcomes, specifically interference or pain intensity (Häuser et al., 2019). A recent meta-analysis (Marin et al., 2021) has also reported weak to no association between ACEs and chronic pain. It appears that not all forms of ACEs are associated with chronic pain in adulthood; rather the ACE should be strong enough to trigger allostatic overload, which may then cause inflammations and desensitize the pain pathways (Beal et al., 2020; Kaleycheva et al., 2021).

### Strengths and clinical implications of the research

The results surprisingly suggested that adverse experiences in childhood may not significantly influence chronic pain outcomes (Gilliam et al., 2019), however SES/income status may have an impact. The ACEs questionnaire did not differentiate maltreatment trauma from general childhood adversity, and clinicians in the field may wish to consider measures that do distinguish these categories when treating chronic pain patients (Dube et al., 2004). It is important for health professionals to collect various types of information during the intake process to gain a comprehensive understanding of patients’ pain conditions. This may include ACEs history, screening for PTSD symptoms and diagnosis, and assessing pain interference, intensity and severity as well as income/education levels. When delivering treatment, healthcare providers should consider utilizing trauma-informed treatment for individuals with ACEs, and be aware that even moderate SES status may impact pain interference and intensity.

### Limitations and future research direction

This research is not without its limitations. Although the ACEs measure is psychometrically sound, it does not measure any duration or severity of abuse, which may have contributed to some non-significant results. Consequently, we were unable to compare whether maltreatment ACEs would induce a different effect on chronic pain outcomes compared to general adversity. In the high ACEs group, individuals may have been repeatedly subjected to heightened stress in childhood (e.g., a combination of emotional neglect and physical abuse and neglect), which may have resulted in allostatic overload and impacted pain outcomes. This was not examined in the current study. Additionally, 89% of participants in the current sample were female, which may limit the generalizability of the findings to male or other gender populations. We also did not detail the ethnicity of the sample in the demographic questionnaire, and because different sex, gender and cultural identities may be associated with distinct factors impacting chronic pain, such as help-seeking behaviors and types of ACEs experienced (Tidmarsh et al., 2022). Furthermore, since the current research is a preliminary study, it did not investigate whether specific PTSS symptoms would impact pain outcomes more or less than others. For example, does hyperarousal impact pain outcomes more than intrusion, and vice versa? Finally, because the overarching trial was an intervention study, possible bias existed with recruitment. Chronic pain sufferers seeking treatment may have biased the number or severity of ACEs, PTSS or even the severity of chronic pain.

Future research is needed to address these gaps in the methodology and generalizability of the current study. Studies should employ more comprehensive ACEs measurements to distinguish maltreatment ACEs from general ACEs. For instance, clinical interviews and verified government records are considered valid and reliable methods for assessing maltreatment ACEs and avoiding recall bias (Marin et al., 2021). The World Health Organization [WHO] (2020) has now published a new version of the ACEs questionnaire, the Adverse Childhood Experiences International Questionnaire (ACEs-IQ), which can be used with a brief clinical interview, detailing six types of ACEs (e.g., exposure to war/collective violence, peer violence, and family violence). Additionally, further studies may utilize more comprehensive PTSD screeners or measurements to identify different PTSS and investigate whether specific symptoms differentially affect chronic pain outcomes and mediate the ACEs-pain relationship. Biomarker measurements (e.g., vagal tone activity and blood pressure) can also be employed to assess whether individuals with ACEs and chronic pain present more activated stress responses than individuals without ACEs.

## Data availability statement

The raw data supporting the conclusions of this article will be made available by the authors, without undue reservation.

## Ethics statement

The studies involving humans were approved by the Bond University Research Ethics Committee. The studies were conducted in accordance with the local legislation and institutional requirements. The participants provided their written informed consent to participate in this study.

## Author contributions

PS and YK contributed to the conception and design of the study. PS oversaw the data collection. YK performed the statistical analysis and wrote the first draft. PS, RS, and JF wrote the sections of the manuscript for publication. All authors contributed to the manuscript revision and approved the final version.

## Footnotes

1
www.psychdata.com
