# Corrigendum: The impact of adverse childhood experiences and posttraumatic stress symptoms on chronic pain

**DOI:** 10.3389/fpsyg.2023.1343626

**Published:** 2023-12-13

**Authors:** Peta Stapleton, Yage Kang, Robert Schwarz, John Freedom

**Affiliations:** ^1^School of Psychology, Bond University, Gold Coast, QLD, Australia; ^2^The Association for Comprehensive Energy Psychology, Bryn Mawr, PA, United States

**Keywords:** chronic pain, pain interference, pain intensity, adverse childhood experiences, post traumatic stress symptoms (PTSS)

In the published article, there was an error. The direction stated in Hypothesis 2 was incorrect. Instead of, “(2) Low ACEs would lead to less severe pain interference and pain intensity compared to no ACEs,” it should be “(2) Low ACEs would lead to more severe pain interference and pain intensity compared to no ACEs.”

A correction has been made to *The impact of adverse childhood experiences and posttraumatic stress disorder on chronic pain, Paragraph 5, Hypothesis 2*. The corrected paragraph is shown below.

Therefore, the present study aimed to examine the level of ACE exposure, categorized as: no ACEs, low ACEs (one to three incidents), and high ACEs (four to 10 incidents). This classification was based on previous findings suggesting that one to three ACEs may result in significantly more chronic pain compared to no ACES (Groenewald et al., 2020; Alhowaymel et al., 2023), and four or more ACEs resulted in a significant increase in risk of chronic pain and PTSS compared to low or no ACEs (Nelson et al., 2021; Alhowaymel et al., 2023). The following hypotheses were proposed:

1. It was hypothesized that high ACEs would lead to more severe pain intensity and interference (a chronic pain profile) compared to no ACEs.
2. Low ACEs would lead to more severe pain interference and pain intensity compared to no ACEs.
3. PTSS would fully mediate the relationship between ACEs and pain outcomes.

The authors apologize for this error and state that this does not change the scientific conclusions of the article in any way. The original article has been updated.

